# Feasibility Study of an Automated Carbohydrate Estimation System Using Thai Food Images in Comparison With Estimation by Dietitians

**DOI:** 10.3389/fnut.2021.732449

**Published:** 2021-10-18

**Authors:** Phawinpon Chotwanvirat, Narit Hnoohom, Nipa Rojroongwasinkul, Wantanee Kriengsinyos

**Affiliations:** ^1^Doctor of Philosophy Program in Nutrition, Faculty of Medicine, Ramathibodi Hospital, The Institute of Nutrition, Mahidol University, Salaya, Thailand; ^2^Department of Computer Engineering, Faculty of Engineering, Mahidol University, Salaya, Thailand; ^3^Institute of Nutrition, Mahidol University, Salaya, Thailand

**Keywords:** carbohydrate counting, computer vision, deep learning, Thai food, eHealth, nutrition

## Abstract

Carbohydrate counting is essential for well-controlled blood glucose in people with type 1 diabetes, but to perform it precisely is challenging, especially for Thai foods. Consequently, we developed a deep learning-based system for automatic carbohydrate counting using Thai food images taken from smartphones. The newly constructed Thai food image dataset contained 256,178 ingredient objects with measured weight for 175 food categories among 75,232 images. These were used to train object detector and weight estimator algorithms. After training, the system had a Top-1 accuracy of 80.9% and a root mean square error (RMSE) for carbohydrate estimation of <10 g in the test dataset. Another set of 20 images, which contained 48 food items in total, was used to compare the accuracy of carbohydrate estimations between measured weight, system estimation, and eight experienced registered dietitians (RDs). System estimation error was 4%, while estimation errors from nearest, lowest, and highest carbohydrate among RDs were 0.7, 25.5, and 7.6%, respectively. The RMSE for carbohydrate estimations of the system and the lowest RD were 9.4 and 10.2, respectively. The system could perform with an estimation error of <10 g for 13/20 images, which placed it third behind only two of the best performing RDs: RD1 (15/20 images) and RD5 (14/20 images). Hence, the system was satisfactory in terms of accurately estimating carbohydrate content, with results being comparable with those of experienced dietitians.

## Introduction

In 2019, type 1 diabetes (T1D) was estimated to affect 1,110,100 persons aged <20 years globally (1). The estimated incidence is 2.07% from 11 tertiary centers in Thailand (2). Briefly, the disease is caused by the progressive destruction of pancreatic beta cells of the immune system. Patients with T1D are dependent on exogenous insulin for glycemic control (3).

Essentially, patients with T1D should estimate the amount of carbohydrates in their foods as precisely as possible using a carbohydrate counting method and administer the appropriate amount of insulin for each meal. Furthermore, patients need to measure their blood glucose levels and record their physical activity to maintain optimal glycemic control. These procedures are part of diabetes self-management (DSM). While patients with T1D should complete DSM regularly to maintain good control of their blood glucose levels, DSM tasks can become a life-long burden.

While carbohydrate counting is crucial for controlling blood glucose levels among patients with T1D, bias, misreporting, and portion size estimation errors are unavoidable for inexperienced (4–6) and even trained persons (7–9). Furthermore, certain patients may find that accurate carbohydrate counting is challenging (and even frustrating) because of inadequate nutritional and/or mathematical knowledge. This challenge may result in suboptimal blood glucose control.

The evolution of a high-resolution digital image sensor, together with the high-performance processor in smartphones, offers a new way to reduce errors in carbohydrate amount estimation. This technology also has the potential to reduce the DSM burden among patients with T1D. With the incorporation of computer vision and deep learning technologies, carbohydrate amounts can be estimated automatically using images of foods taken by a smartphone in a real-time fashion.

Several estimation systems have been developed to overcome problems of inaccuracy (10–12) that demonstrate comparable results with estimations made by experienced dietitians. In addition, some systems have been equipped with functions for recording individual health status on a daily basis, including food intake, physical activities, blood glucose levels, and insulin dosage. This information can also be exported to healthcare providers. Such features can reduce the burden of diabetes care process. However, the direct application of these systems to include Thai Foods can be challenging due to differences in visual appearance, cultural food preparation practices, cooking methods, and local ingredients, amongst others.

Compared with Western foods, carbohydrate counting in Thai foods is more challenging (13–15). The visual appearance of similar foods can vary because of the customization of food ingredients. For instance, several fruits, such as mango, tamarind, pomelo, star gooseberry, and carambola, can be used along with lime juice to produce acidic flavors in Thai foods, thus providing a significant amount of carbohydrates. Even for foods with the same visual appearance, carbohydrate content can vary depending on the cooking method. Consequently, Thai dietitians must estimate carbohydrate content based on a standard recipe and then modify the carbohydrate content by taking into account the visible or known ingredients in each dish (16).

To the best of our knowledge, while there have been many attempts to develop a system for Thai food recognition (17–19), no system has aimed at estimating carbohydrate content in Thai foods. The objective of this study, therefore, was to develop a carbohydrate estimation system using single images of Thai foods taken using smartphones. Moreover, in this study, system estimation results were validated against those of experienced dietitians to ensure that the newly developed system is practical and accurate.

## Materials and Methods

### System Outline

The system proposed by this study was based on the traditional approach for image-assisted dietary assessment (20–23) entailing food recognition, food volume estimation, and nutrient calculation from a food composition database. The system was designed to use only a single RGB-color image for determining the carbohydrate content of specific individual foods. The system consisted of three independent algorithms, namely, the convolutional neural network (CNN)-based object detector, segmentation unit, and neural network regression-based weight estimation unit. The state-of-the-art YOLOv4-tiny (24) was employed for the object detection task. Transfer learning was used to retrain a Microsoft Common Objects in Context (MS COCO) pre-trained model for the newly developed dataset. Darknet was used initially for training, and the trained model ran on OpenCV's dnn module after being trained well. The system demonstrated Top-1 accuracy at 80.9% for food recognition in the test dataset. The classic GrabCut (25) was used for image segmentation.

The regression model consists of an input layer, three dense hidden layers with 100, 50, and 25 nodes, and 1 node of the dense output layer. Rectified linear units were used for the activation function between each layer, and mean squared error was used for loss function with Adam optimization. An 11 × 1 input layer, matching the food item area and the location of it, referring to values of xmin, ymin, xmax, ymax, the reference object area, reference object location of the same image, and shooting angle, was used for training spoon-corrected models. A 6 × 1 input layer was used for training no-spoon-corrected models using the same data, but reference object information was omitted. For each food class type, the dataset was split into 80% for training and 20% for testing from three different angles equally. The weight estimation unit selected the spoon-corrected models when the spoon was present in the input image.

**Figure 1:**
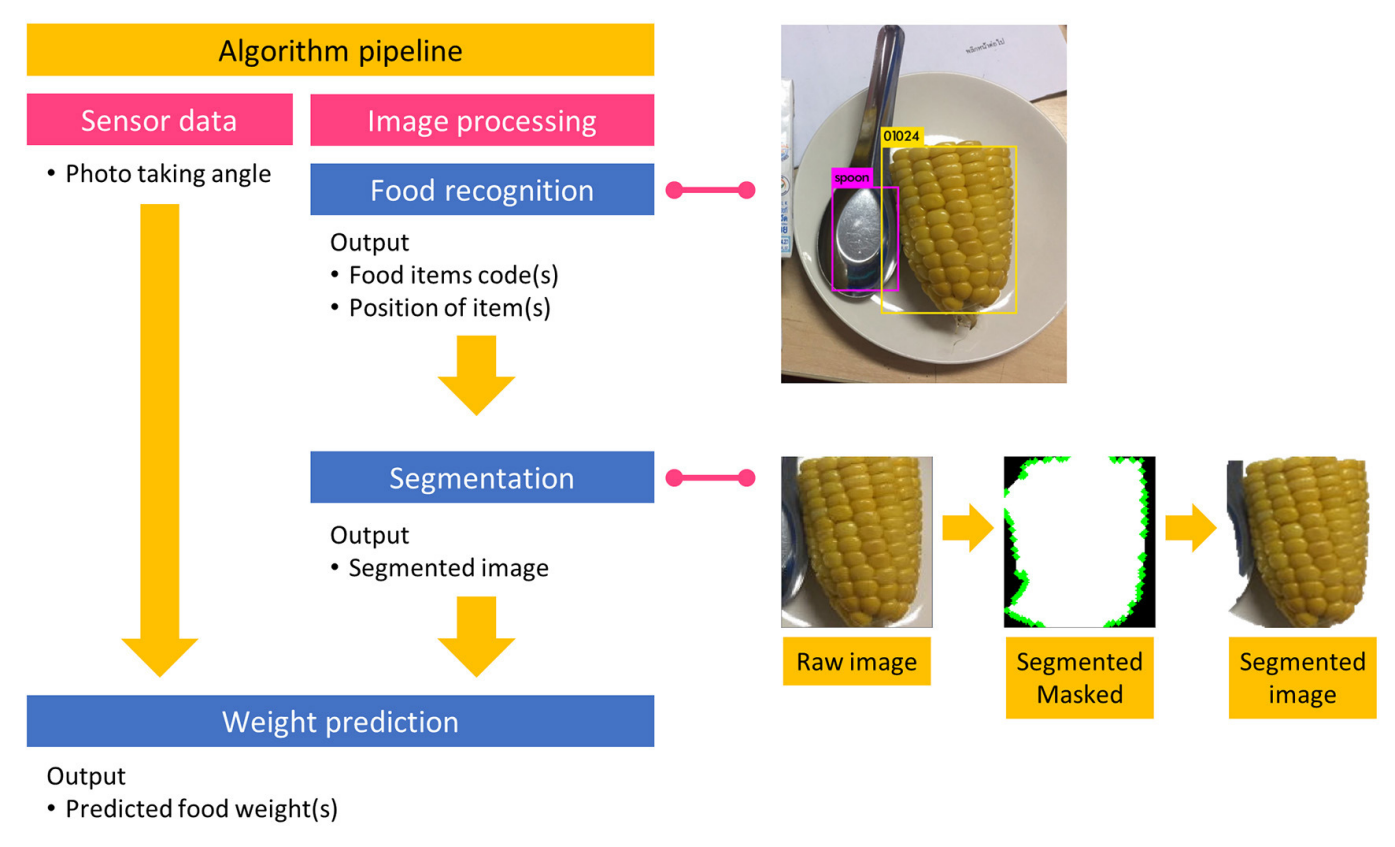
Overview of the system.

**Figure 2:**
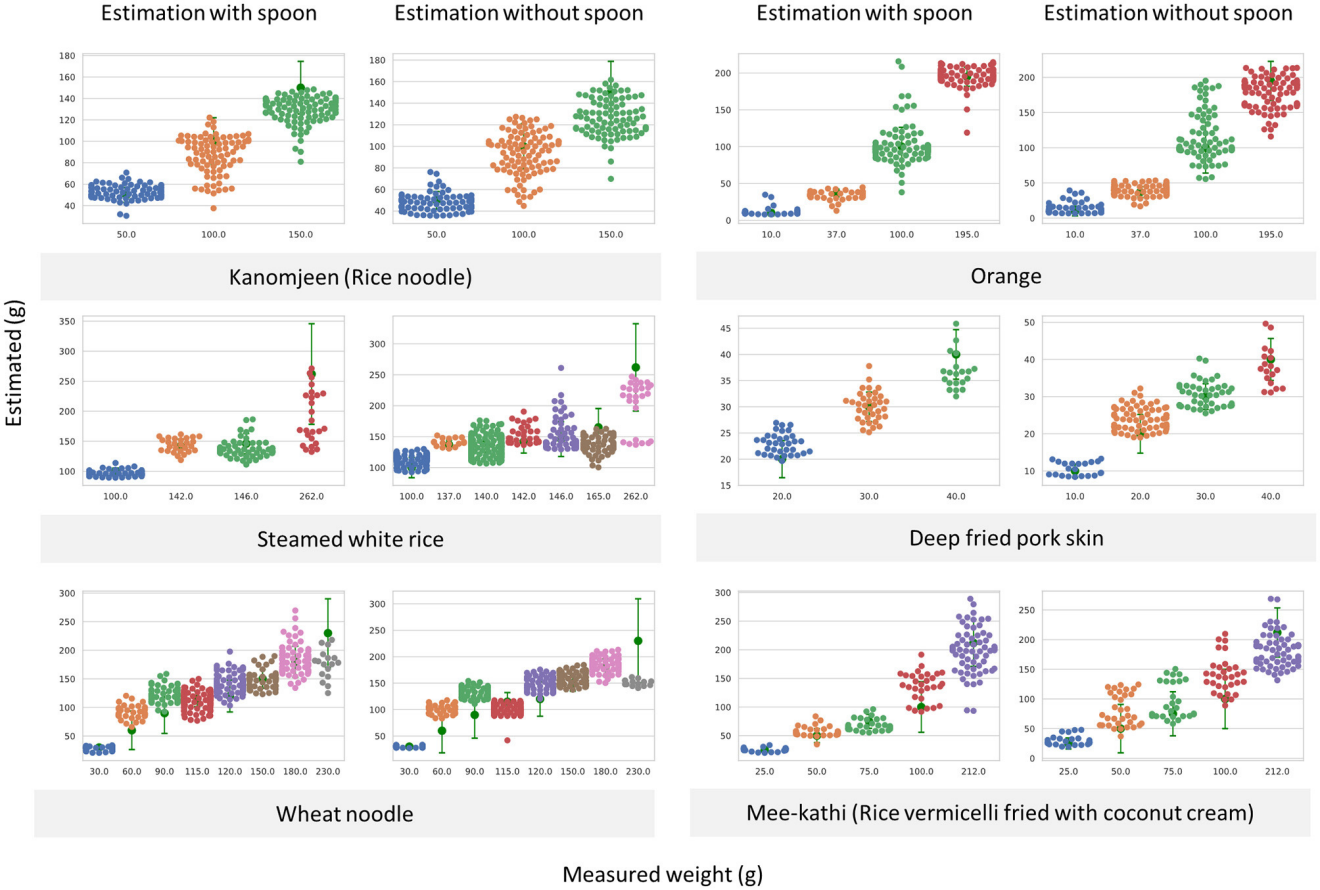
Swarm plots illustrating measured weights and weight estimation results with and without using spoons as reference objects from the test dataset. This level of error could produce the RMSE of calculated carbohydrate <10 g for all classes.

After the image acquisition process, an object detector identified the areas of food components in each image. The food code and region-of-interest (ROI) were sent to the segmentation unit wherein non-food regions were removed automatically. Incorporating the angle at which each photo was taken, the segmented food component images were transferred to the weight estimation unit. Finally, the estimated weight of each food component was sent to the food composition computational unit to determine nutritive values and the amount of carbohydrates. The summation of all nutrients for each food component was used to report the nutritive values of each food image. An overview of the system is illustrated in Figure 1. Previous reports have found that children living with T1D can tolerate an estimation error of up to 10 g of carbohydrates without any effect on postprandial blood glucose. However, an estimation error beyond 20 g of carbohydrates may have deleterious effects on postprandial blood glucose (26). Consequently, the system aimed to have an estimation error of <10 g of calculated carbohydrates as measured by root mean square error (RMSE) when compared with the measured food weights for all items in the test dataset. Selected weight estimation results from the test dataset are illustrated in Figure 2.

### Building the Thai Food Image Dataset

To the best of our knowledge, a Thai food image dataset with measured food weights was not available at the time of this study. Consequently, the dataset was built from scratch. According to a previous report (27), 80% of carbohydrates consumed by the Thai population frequently come from only 93 food items as determined by the INMUCal-Nutrients software (28). Hence, these items were included in the newly developed dataset. For expanding coverage in real life, some traditional Thai desserts, favorite tropical fruits, and vegetables, as well as meats, were also included in the dataset. The final image dataset contained 175 food item classes. The selected foods, which were similar to their descriptions in the INMUCal database (cooking methods, appearance, etc.), were purchased from two to three local food vendors and prepared into ready-to-eat forms. Some of the foods were diced, chopped, or ground to change their physical appearance before being placed on low-reflective, white, flattened 9-inch plates or low-reflective, white 6-inch bowls. For some foods, such as those packed in ready-to-eat containers like foam boxes, photos were taken in each food's original container and afterwards transferred to a plate. Generally, no part of the food was covered by another food. However, for some menus consisting of rice topped with cooked meat, curry, or an opaque sauce, they were arranged to have photos taken both with and without toppings. For each type of food, the portion sizes of food items were gradually increased by adding 1 or ½ exchange unit for a large portion-sized food. All the food portions were measured using a digital kitchen scale and rounded to the nearest integer, for instance, 63.4 and 55.5 g were rounded to 63 and 56 g, respectively. Various shapes of tablespoons were randomly placed on plates along with the foods to be used as reference objects.

**Figure 3:**
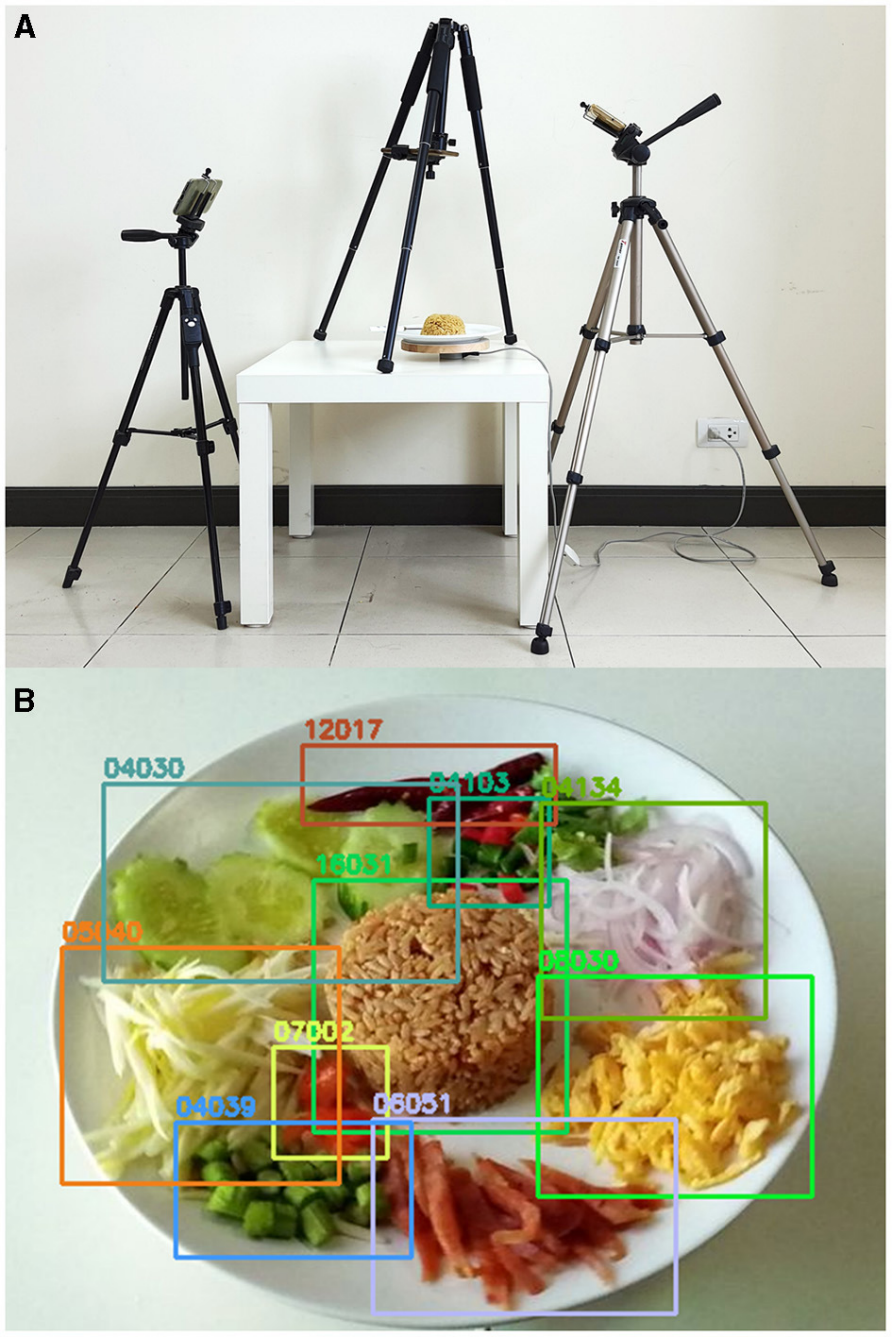
**(A)** Image acquisition setup for building the image dataset and **(B)** demonstration of how a dietitian annotated the ingredients of Khao-kluk-kapi (fried rice with shrimp paste), including fried rice with shrimp paste (16031), thin sheets of fried egg (08030), Kun-chieng (dried sweet pork sausage) (06051), yardlong bean (04039), unripe mango (05038), cucumber (04030), dried chili (12017), bird's eye chili (04103), and dried shrimp (07002). The five-digit code is the INMUCal food code.

**Figure 4:**
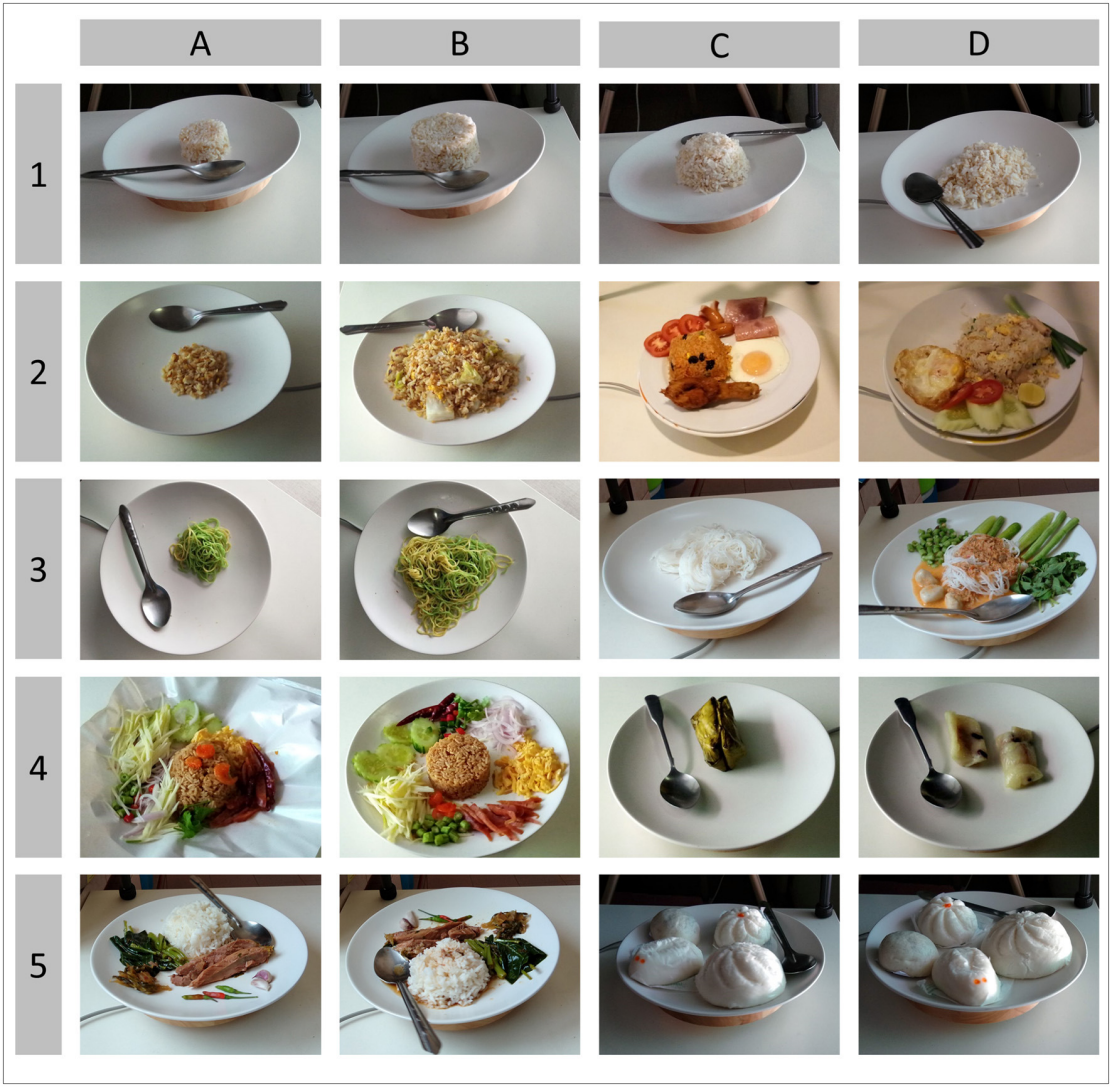
Examples of Thai food images in the newly developed dataset; **(A1:D1)** represent gradual increases in the portion size of steamed white rice; **(A2:D2)** represent variation in visual appearance of **(A2:B2)** fried rice entailing plain fried rice, **(C2)** fried rice with ketchup and raisins, and **(D2)** fried rice as provided by food vendors; **(A3:B3)** are wheat noodles in gradually increasing portion sizes; **(C3)** is plain Kanomjeen (rice noodle), and **(D3)** is Kanomjeen topped with Namya-pla (ground fish in spicy coconut milk curry) and vegetables; **(A4)** is food vender prepared Khao-kluk-kapi (fried rice with shrimp paste), while **(B4)** is the rearranged form of the food as recommended for carbohydrate counting. **(C4)** is Kaotom-mud (glutinous rice steamed with coconut milk) wrapped in a banana leaf container, and **(D4)** is the unwrapped form of the dessert; **(A5:B5)** are steamed white rice with Ka-moo-palo (pig leg in a sweet stew), and **(C5:D5)** are various shapes of steamed buns, representing relocation of food items due to plate rotation.

White plates or other food containers were individually placed on top of an electric-powered rotating plate, which was then placed on top of a low-reflective white table. Three Android smartphones were attached to three camera tripods to take photos from three different angles (30°, 60°, and 90° from the tabletop). While the rotating plate was operating, burst shots were taken to capture pictures simultaneously, resulting in a series of multi-angle food images. After the removal of low-quality or duplicated images, the remaining images were resized into 800 × 800 pixels using Lanczos algorithms to allow the annotators to function conveniently in any device. All visible food components were annotated separately in the same fashion as that for Thai dietitians, and only the head (bowl) part of a spoon was annotated as a reference object. Image-acquisition setup and examples of annotation are illustrated in Figure 3. The newly developed dataset contained 256,178 ingredient objects from 175 ingredient categories in 75,232 images. This image dataset was used for both object detector and weight estimator unit training together with 1,800 non-food images. Figure 4 displays example images from the newly developed dataset.

### Reliability Test and Statistical Analysis

To assess the accuracy of the newly developed system, estimated results (Estimated) were compared with measured weight (Measured) and estimations by experienced registered dietitians (RDs). Since there are no valid criteria to directly compare accuracy, a set of statistical analyses was used to determine the accuracy of carbohydrate estimation, including RMSE, Pearson's correlation coefficient (*r*), Lin's concordance correlation coefficient (Rc), paired *t*-test, and Bland–Altman plot. A two-sided *p* < 0.05 was considered statistically significant. The Dupont and Plummer method was used to determine sample size estimation for dependent measurement values. When the mean difference (δ) of carbohydrate estimation between two methods was set to 10 g, and the standard deviation of difference (σ) in the response of matched pairs, as obtained from literature, was 10.12 (11), only 13 images were required for testing with power = 0.9 and α = 0.05. However, 20 known-weight food images were used in the testing. Twenty food menus were randomly selected from the image dataset. These foods were purchased from local food vendors and prepared in ready-to-eat forms with a variety of portion sizes. Food photos were taken freely (5–10 images for each food) that resulted in 300 images for validation testing. Finally, 20 images were randomly selected from the validated dataset, containing 48 food items, and used for both dietitians and the newly developed system to estimate portion sizes. Instead of using the image acquisition process on smartphones, which can repeat several times until the object detector can recognize all food components, the images were sent to the system directly to mimic dietitians performing the method. Eight RDs from Theptarin Hospital agreed to participate in this study. Six RDs were also certified diabetes educators by the Thai Association of Diabetes Educators. Five RDs had performed image-assisted dietary assessment in a previous study (27). Because most of the RDs estimated in exchange units, the results were converted back to weight in grams for identified foods according to the Thai Food Exchange List (29) or the official Carbohydrate Counting guidebook (16). Carbohydrate content and nutritive values were calculated based on the same principles unless the RDs specified the amount of weight, carbohydrates, or nutritive values.

## Results

The system could detect 36 of the 48 food items (75%) among 20 food images. Missing items included three leafy vegetables (cucumbers and white jelly fungus) and eight meat products [Kun-chieng (Chinese sausage/dried sweet pork sausage), boiled chicken breast and blood, cuttlefish, fish strips, and boiled eggs], all of which do not significantly contribute to the amount of carbohydrate in foods.

**Figure 5:**
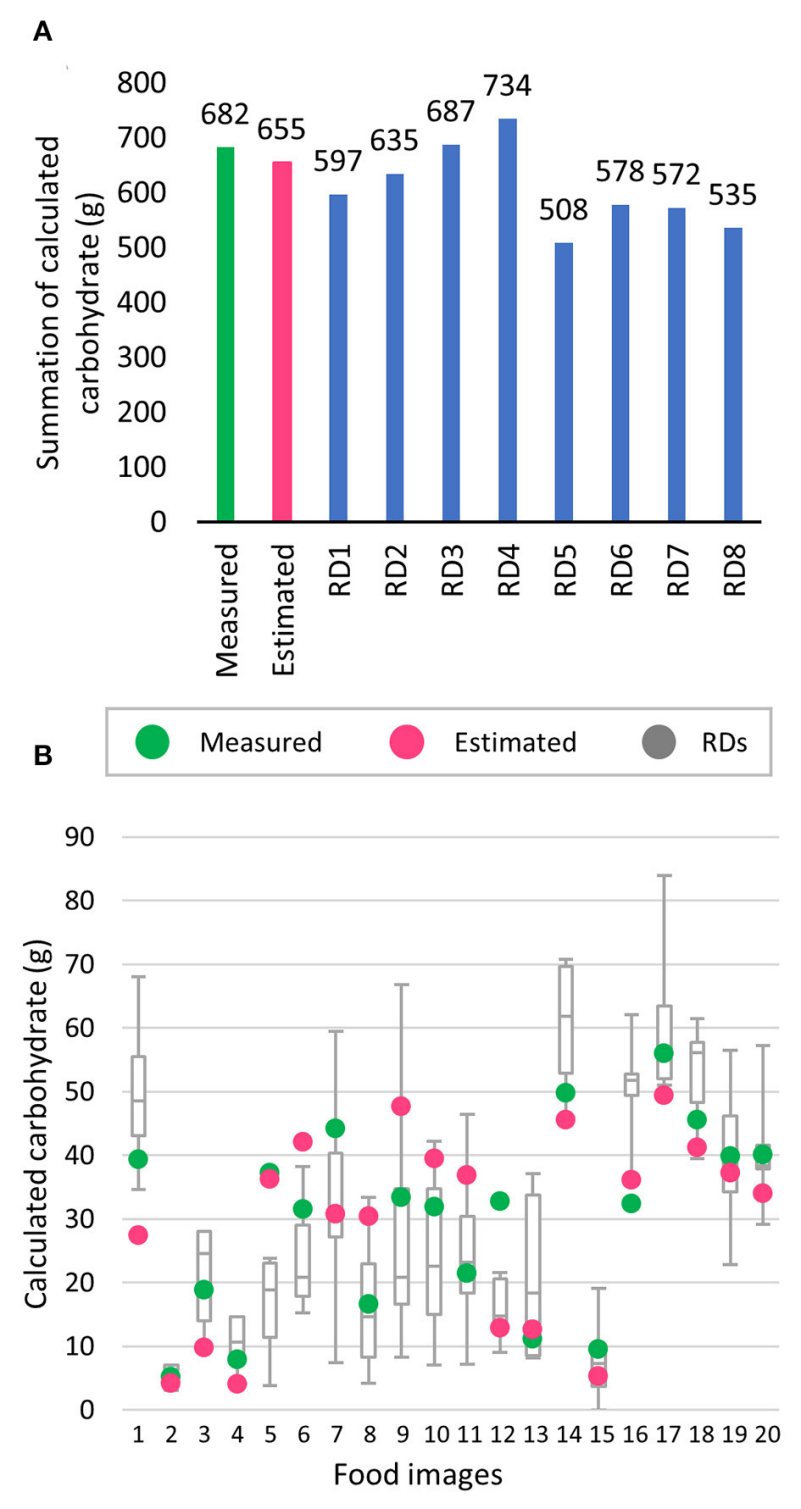
Comparison of calculated carbohydrate between measured weight (green), estimated weight (pink), and estimated results from eight registered dietitians (RDs); **(A)** Summation of carbohydrates; **(B)** scatter plot of calculated carbohydrate of each image for Measured (green), Estimated (pink), and box and whisker plot represent the estimation results from eight RDs.

For the 20 images, overall, summations of the calculated carbohydrate content for measured and estimated were 682 and 655 g (4%), respectively, while the nearest, lowest, and highest estimations by RDs were 687 (0.7%), 508 (25.5%), and 734 g (7.6%), respectively. All the summations of calculated nutrients are illustrated in Figure 5A. For each image, the system had an estimation error of <10 g of calculated carbohydrates for 13 images, which ranked in third place, behind RD1 (15 images) and RD5 (14 images). The scatter plots of raw values are illustrated in Figure 5B.

The RMSE of the calculated carbohydrate content between Measured and Estimated was 9.4, while the lowest and highest RMSE by the RDs were 10.2 and 19.5, respectively. Lin's concordance correlation coefficient (Rc) was 0.79, and Pearson's correlation coefficient (*r*) was 0.8. These results placed the system in second place, only behind the RD5 who had Rc = 0.79 and *r* = 0.83.

For group mean comparisons, the difference between the means of the calculated carbohydrate content of Measured (30.3 ± 14.8 g) and Estimated (29.2 ± 15.3) was small enough that the paired *t*-test could not detect the difference as indicated by a *p*-value of 0.625, whereas the paired *t*-test was able to detect the difference between the highest (36.7 ± 19.7) of RD4 and the lowest (25.4 ± 16.5) of RD5 with *p*-values of 0.047 and 0.03, respectively. Statistical analysis results are given in Table 1.

**Table 1:** Statistical results for the estimation of error, agreement, and relationship as measured by RMSE, Rc, and *r*, respectively, between estimated and RDs of carbohydrate against measured in the validated dataset.

	**RMSE**	**Rc**	***r***	**Mean ± SD**	***t*-test**
Estimated	9.4	0.79	0.80	29.2 ± 15.3	0.625
RD1	10.6	0.75	0.75	29.8 ± 15.8	0.868
RD2	14.4	0.69	0.74	31.7 ± 21.7	0.658
RD3	12.3	0.71	0.74	34.4 ± 17.6	0.137
RD4	14.7	0.65	0.73	36.7 ± 19.7	0.047^*^;
RD5	10.2	0.79	0.83	25.4 ± 16.5	0.030^*^;
RD6	11.8	0.76	0.80	28.9 ± 19.9	0.625
RD7	19.5	0.56	0.66	28.6 ± 26.3	0.719
RD8	10.5	0.77	0.80	26.8 ± 16.7	0.141

*RMSE, root mean square error; Rc, Lin's concordance correlation coefficient; r, Pearson's correlation coefficient; t-test, paired t-test p-value*.

* *Statistically significant*.

**Figure 6:**
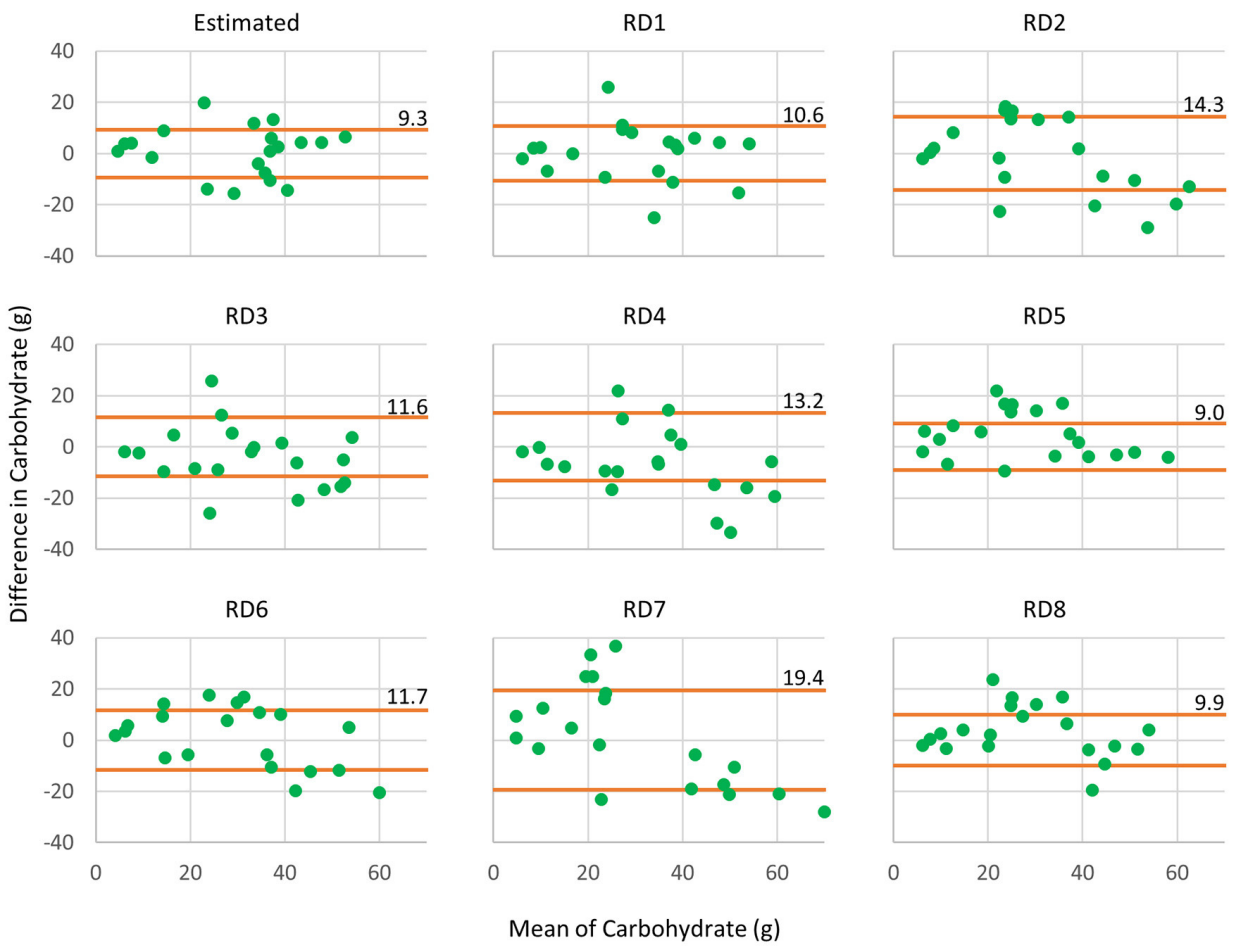
Bland–Altman plots show agreement between calculated carbohydrate content of Measured, Estimated, and RDs. The orange lines are limits of agreement between other methods against Measured.

The Bland-Altman plots reveal that the system could produce a narrow limit agreement for calculated carbohydrate content, as illustrated in Figure 6. According to these results, the system could provide comparable carbohydrate estimation compared with the RDs.

## Discussion

In this study, a system was designed to perform carbohydrate counting automatically using only a single image without any user interaction. The testing environment mimicked a real-world scenario where several food items might be present in the same dish and the portion size along with the visual appearance of each item may vary. Sources of variation include food preparation, photo taking angles, and the distance between foods and the smartphone. The system was tested using several statistical means to ensure the robustness of carbohydrate estimation accuracy. The results are convincing since the system resulted in estimates comparable with those of experienced RDs.These accurate results also support the use of this system in a real-world situation by people living with T1D.

Traditionally, image-assisted dietary assessment is composed of food recognition, segmentation, food volume estimation, and calculation of nutritive values using a food composition table (20–23, 30). The overall accuracy of the system is dependent on the accuracy and performance of correspondent algorithms to handle each step, which has been improved continuously over time through the efforts of many researchers (31–33). With the aid of cutting-edge technologies, such as deep learning and CNNs, some steps in the traditional method can be bypassed with better accuracy. Two pioneering studies that used CNNs to predict food volume from just a single RGB image have achieved fascinating results (34, 35). The systems were trained using RGB images and their corresponding depth information, and then a depth map was applied to predict food volumes. In addition to volume estimation, CNNs also have the potential to predict nutritive values directly without utilizing a food composition table (36), which could be accomplished by training the system with nutrient content annotated food images.

Applying these advanced approaches to the situation in Thailand is more challenging. One major barrier to applying these approaches is the need for a large dataset for deep learning training. Acquiring a large image dataset with depth images or analyzed nutritive values is costly and may not be suitable under limited resource conditions. For this reason, the proposed system still relied on the multistep traditional method for image-assisted dietary assessment but was re-implemented with modern techniques, such as deep learning.

YOLOv4-tiny was selected when considering trade-offs between detection accuracy and efficiency of resource usage. GrabCut could handle segmentation sufficiently when the interested area contained only a single object and was surrounded by a monotonous background to predict results from the object detector. Volume estimation by 2D pixel density and calibration with a reference object is an unsophisticated yet more feasible choice. Two previous reports (22, 37) have applied this method and demonstrated acceptable results in their datasets consisting of 15 and 20 classes of solid food. The former report described implicitly how to estimate the volume and measure the accuracy of estimation, but the latter used one of three formulas based on the shape of foods to estimate food volume and corrected with the size of a One-Yuan coin. Instead of constructing a new volume estimated formula, simple feedforward neural networks were selected and trained with shooting angle, object area, object location, reference object area, and reference object location, and measured weight was treated as a latent variable.

Building a new image dataset with weight annotation could overcome two additional problems. While Microsoft COCO (38), PascalVOC (39), and ImageNet (40) are dominantly public datasets for image classification, they contain a limited number of food classes. While Food-101 (41), UEC Food100/256 (42, 43), and VIREO Food-172 (44) are considered large, high-quality image datasets for food recognition, they do not include volume information and are also limited in the number of Thai foods included. Food specific density is equally important as measured ground truth food volume for the volume estimation approach, but relevant data are not available in the current version of the INMUCal database. Furthermore, a recent report raised a concern about the accuracy of the volume to weight conversion method or *vice versa* (45). Both of which could be eliminated with ease by creating a new dataset with the weight of food instead of volume.

Due to several reasons, the results here cannot be compared directly with results from prior studies. Our system was developed based on common practices of carbohydrate counting by Thai dietitians, in which foods with several food items are counted separately and the amount of carbohydrate is modified based on visible or known components. If food items are already mixed into a prepared food dish by a vendor, a client must separate each item as much as possible (16). For example, the amount of carbohydrates in a typical fried rice dish does not differ substantially from that of steamed rice (29 vs. 30 g per 100 g). However, when the fried rice is mixed with raisins, the amount of carbohydrates should be increased by 1 g for each raisin used in the dish. To comply with this tradition, the fried rice and all raisins in the image must be annotated separately. When the system can detect one item of fried rice and five raisins from a total of 10 raisins as presented in the image, this results in 55% detection accuracy (6 from 11 items), while the carbohydrate estimation error is 5 g. This situation makes comparing recognition accuracy with results from prior studies challenging. Furthermore, the newly developed system was trained with foods that are consumed daily in Thailand, and different from Thai foods available in other countries and presented in other datasets. Even though some Thai food menu items may have the same name, the ingredients, cooking methods, and visual appearance may be different. Moreover, methods to validate estimation accuracy among previous studies vary greatly. Three recently published review articles on image-based dietary assessment indicate a great variety of accuracy measurement procedures (31–33).

Estimation errors made by the RDs were noticeable, since not all of them were familiar with all of the foods, and not all the RDs could remember exactly the exchange weights for all foods listed in official resources. These limitations were evident especially for glutinous rice desserts that had considerable estimation errors, as displayed in food picture numbers 7, 9, and 10 in Figure 5B, which represents Kaotom-mud (glutinous rice steamed with coconut milk), Khaoniao-sang-khaya (glutinous rice with custard), and Khaoniao-na-kung (glutinous rice with sweet, dried shrimp), respectively. Inaccuracies were made in terms of portion estimation, as well as a large variation in guessing hidden sugars in these desserts, largely due to difficulty in correctly guessing unfamiliar foods. RD4 and RD5 were affected especially by these limitations, which resulted in significant over- and under-estimation of mean values, respectively, as shown in Table 1. The problems can be lessened with the aid of a nutrient calculation mobile application.

While the system was satisfactory in terms of accurately estimating carbohydrate content, it can only produce a fair level of satisfaction for protein and fat estimation. Because the new food dataset in this study was aimed at carbohydrate counting, it contained only a limited number of items, low variety in terms of visual appearance, and limited portion sizes for both meats and vegetables. These issues can affect the ability of an object detector to perform effectively. Furthermore, when the food container is a patterned plate or made with a highly reflective material, this can also interfere with the performance of the object detector as well as GrabCut.

Even though the trained dataset was constructed from three angles, and an electronic rotating plate was used to vary food item appearance, camera distance was not changed as it was the recommended distance for applying the system. Hence, the system could not detect food items behind other food items, even when some parts of a food item were present in the image. However, the object detector could occasionally detect a food item in the background, but the size of this food would be smaller than its actual size. Furthermore, presenting more than one food object without enough space between each object can affect GrabCut in terms of foreground extraction and subtraction of the background correctly. These problems could potentially introduce inevitable errors in the system. However, even though the system can perform with several limitations, user awareness of these can overcome them, such as by separating and rearranging food items on the monotonous background plate, placing spoons beside foods, and keeping the distance between foods and a mobile phone at around 30 cm.

## Conclusion

This study provides evidence for the feasibility of a deep learning-based carbohydrate estimation system using Thai food images. The system was able to achieve estimation results comparable with those of the dietitians in this study. Further studies are warranted to test the effectiveness of this newly developed system in aiding DSM and improving glycemic control in people living with T1D and, thus, lessening the burden of counting carbohydrates in their food.

## Data Availability Statement

The raw data supporting the conclusions of this article will be made available by the authors, without undue reservation.

## Author Contributions

PC: conceptualization, software development, formal analysis, and writing of the first draft of the manuscript. WK: conceptualization, organization, review, and critique of the manuscript. NH: software development, review, and critique of the manuscript. NR: review and critique the statistical analysis and manuscript. All the authors have read and agreed to the published version of this manuscript.

## Funding

PC was a recipient of the Institute of Nutrition PhD Scholarship and the Research Assistantship from the Faculty of Graduate Studies, Mahidol University Academic Year 2019.

## Conflict of Interest

The authors declare that the research was conducted in the absence of any commercial or financial relationships that could be construed as a potential conflict of interest.

## Publisher's Note

All claims expressed in this article are solely those of the authors and do not necessarily represent those of their affiliated organizations, or those of the publisher, the editors and the reviewers. Any product that may be evaluated in this article, or claim that may be made by its manufacturer, is not guaranteed or endorsed by the publisher.
